# The Application of Impedance Spectroscopy for *Pseudomonas* Biofilm Monitoring during Phage Infection

**DOI:** 10.3390/v12040407

**Published:** 2020-04-07

**Authors:** Grzegorz Guła, Paulina Szymanowska, Tomasz Piasecki, Sylwia Góras, Teodor Gotszalk, Zuzanna Drulis-Kawa

**Affiliations:** 1 Department of Pathogen Biology and Immunology, Institute of Genetics and Microbiology, University of Wroclaw, 51-148 Wroclaw, Poland; grzegorz.gula@uwr.edu.pl (G.G.); sylwiagoras@gmail.com (S.G.); 2 Department of Nanometrology, Faculty of Microsystem Electronics and Photonics, Wroclaw University of Science and Technology, 50-372 Wroclaw, Poland; paulina.szymanowska@pwr.edu.pl (P.S.); tomasz.piasecki@pwr.edu.pl (T.P.); teodor.gotszalk@pwr.edu.pl (T.G.)

**Keywords:** *Pseudomonas aeruginosa*, phage LUZ19, biofilm, quartz tuning forks, impedance spectroscopy

## Abstract

Bacterial biofilm prevention and eradication are common treatment problems, hence there is a need for advanced and precise experimental methods for its monitoring. Bacterial resistance to antibiotics has resulted in an interest in using a natural bacterial enemy—bacteriophages. In this study, we present the application of quartz tuning forks (QTF) as impedance sensors to determine in real-time the direct changes in *Pseudomonas aeruginosa* PAO1 biofilm growth dynamics during *Pseudomonas* phage LUZ 19 treatment at different multiplicities of infection (MOI). The impedance of the electric equivalent circuit (EEC) allowed us to measure the series resistance (Rs) corresponding to the growth-medium resistance (planktonic culture changes) and the conductance (G) corresponding to the level of QTF sensor surface coverage by bacterial cells and the extracellular polymer structure (EPS) matrix. It was shown that phage impacts on sessile cells (G dynamics) was very similar in the 10-day biofilm development regardless of applied MOI (0.1, 1 or 10). The application of phages at an early stage (at the sixth h) and on three-day biofilm caused a significant slowdown in biofilm dynamics, whereas the two-day biofilm turned out to be insensitive to phage infection. We observed an inhibitory effect of phage infection on the planktonic culture (Rs dynamics) regardless of the MOI applied and the time point of infection. Moreover, the Rs parameter made it possible to detect PAO1 population regrowth at the latest time points of incubation. The number of phage-insensitive forms reached the level of untreated culture at around the sixth day of infection. We conclude that the proposed impedance spectroscopy technique can be used to measure the physiological changes in the biofilm matrix composition, as well as the condition of planktonic cultures in order to evaluate the activity of anti-biofilm compounds.

## 1. Introduction

Bacterial biofilm is a complex and dynamically changing structure responding to the external environment. Microbial cell aggregates are surrounded by complex polymer substances (matrix) composed of proteins, polysaccharides, eDNA (extracellular DNA), glycolipids and fatty acids [1]. Bacteria inside the biofilm are not synchronized in terms of metabolic activity, and biofilm structure is highly heterogeneous in this respect [2,3]. The protection against unfavorable environmental conditions can be implemented by the extracellular polymer structure (EPS) production, increasing significantly the resistance to chemical compounds and the immune system response [4]. Biofilm is a long-term strategy for bacterial survival under stress conditions. Cells embedded in the consortia cause biomaterials dysfunction, chronic infections, are highly insensitive to treatment and pose a threat in industry or are responsible for biofouling [5]. The high insensitivity of biofilm to antibiotics forces the search for alternative treatment solutions. Bacteriophages turn out to be a convenient weapon against both planktonic and sessile forms of bacteria [3,6,7]. To evaluate the complex and sublime interactions of antibacterials and biofilm cells it was necessary to develop various measurement techniques. 

Currently, there are several methods allowing the characterization of biofilm components. The colony count (CFU/mL) of sessile cells colonizing biofilm structure is commonly used. The crystal violet (CV) assay is used for biomass measurement because the dye is non-specific and interacts with negatively charged moieties of cells and biofilm matrix [8]. Other staining techniques are more specific showing the dye interaction with different biofilm elements. The commonly used Live/Dead BacLight kit composed of SYTO^®^ dyes with orange acridine or propidium iodide (Merck Millipore, Burlington, MA, USA), distinguishes live and dead cells [9]. Resazurin (Alamar Blue), XTT (2,3-bis-(2-methoxy-4-nitro-5-sulfophenyl)-2H-tetrazolium-5-carboxanilide) and TTC (2,3,5-triphenyl-2H-tetrazolium chloride) are another group of markers for determining metabolically active cells. Calcofluor white or FITC-labeled lectins are used to stain the glycoconjugate fraction of biofilm [10]. FITC also interacts with the amino residues of proteins and amino sugars [11,12]. For fast determination, it is possible to use Nile Red staining protocol [10]. These dyes can be detected spectrophotometrically, but also fluorometrically using microplate readers or CLSM microscopy [13]. 

Visualizations of biofilm development and its eradication by antibiotics, phages or other combined therapies are an inseparable group of methods used in biofilm research. Microscopy techniques based on transmission electron microscopy (TEM), scanning electron microscopy (SEM), atomic force (AFM) or confocal microscopy are often used [14,15]. However, this group of methods is quite expensive and requires the use of sophisticated microscopy tools and material preparation techniques.

There is also an interesting method for biofilm measurement based on interferometry spectroscopy allowing us to determine the changes in the diffusion rate through the biofilm matrix [3,7,16,17]. 

Each method used for biofilm monitoring has both advantages and limitations. In most cases, they are based on mandatory material preparation techniques, and do not allow measurements of long-term biofilm growth dynamics under the culture conditions. Monitoring of changes in the biofilm structure (EPS and cells) in real-time is particularly difficult. In this paper, we present the application of the impedance spectroscopy (IS) based on the application of quartz tuning forks (QTFs) as an impedance sensor. We have already published the physical background of QTF system applications in biofilm mass measurements [15,18]. 

In this study, we present our impedance spectroscopy set up for the monitoring of electrical properties of biofilm structure and bacterial culture. Measuring conductance and, we were able to detect the changes in biofilm development as well as in planktonic cells propagation. The application of QTF impedance systems were tested and verified on a *Pseudomonas aeruginosa* PAO1 culture model treated with *Pseudomonas* lytic phage LUZ19.

## 2. Materials and Methods 

### 2.1. Bacterial Strain and Phages 

*Pseudomonas aeruginosa* PAO1 (ATCC 15692) was used as a model of biofilm-forming bacteria. Bacterial cells were stored at −70 °C in Trypticase Soy Broth (TSB, Becton Dickinson and Company, Cockeysville, MD, USA) supplemented with 20% glycerol (U.S. Merck Corporate Headquarters, Kenilworth, NJ, USA). For the experiments, strains were refreshed on Trypticase Soy Agar (TSA, Becton Dickinson and Company, Cockeysville, MD, USA) at 37 °C for 18 h. *Pseudomonas* LUZ19 podovirus equipped with polysaccharide depolymerase was kindly provided from the collection of the Laboratory of Gene Technology, KU, Leuven, Belgium.

### 2.2. Bacterial Growth Measured by the Colony Count

*P. aeruginosa* PAO1 culture was incubated at 37 °C in 1.5 mL of TSB medium with immersed quartz tuning forks (QTF). The biofilm and planktonic growth dynamics were measured by the classical colony forming units (CFU/mL) at specific time points: at 6, 12, 24, 48, 72, 96, 144, 192, 216 and 240 h of the experiment. QTFs covered with biofilm were transferred to eppendorf tubes containing 150 µL of PS (physiological saline, Avantor Performance Materials, Gliwice, Poland). To liberate cells from the biofilms and to measure the cell counts, biofilm was disintegrated using the ultrasonic cleaning bath USC300TH with 45 kHz frequency and 80 W power for 25 min for all sensors (VWR International Ltd., Lutterworth, Leicestershire, UK), released cells were plated on Trypticase Soy Agar plates (Becton Dickinson and Company, Cockeysville, MD, USA) and the colony count was evaluated after 18 h incubation at 37 °C. The colony count was done for phage treated and untreated samples. 

### 2.3. Phage Treatment

*Pseudomonas* phage LUZ19 filtrate at 10^9^ pfu/mL (plaque forming units) was diluted to obtain a corresponding MOI 0.1, 1 and 10 used from biofilm treatment at different time points: 6, 48, 72 h. Phages were suspended in TSB medium in 1.5 mL into which sensors with adhered biofilm were transferred. Phage LUZ19 at a concentration of 10^9^ pfu/mL and TSB alone were used as negative controls (no biofilm). All experiments were performed three times in at least 4 technical replicates.

### 2.4. Bacterial Biofilm Growth Measured by the Impedance Method

As was reported before [18], the Quartz Tuning Forks (QTFs) may be used as the impedance sensors for biofilm growth monitoring. The measured object may be the impedance sensor which electric impedance change with the changes of the environment in which it is placed.

**Figure 1:**
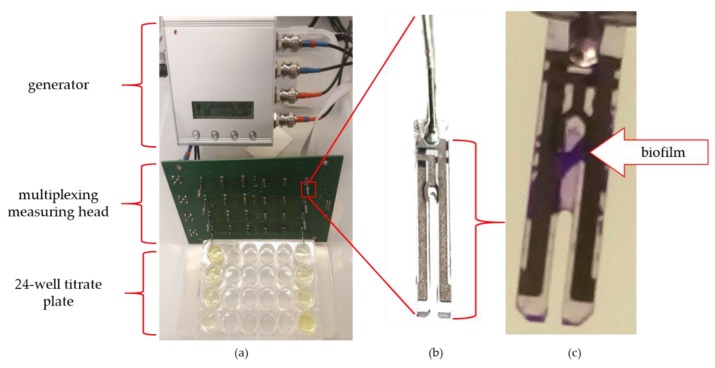
The measurement setup: (**a**) measurement system; (**b**) quartz tuning fork (QTF) senor; (**c**) biofilm covered QTF surface detected by crystal violet (CV) staining.

To perform the impedance spectra, the IMP-STM32 impedance analyser was used [19] connected to a dedicated multiplexing measuring head with sockets for 24 QTF sensors (Figure 1). Such a setup allowed for the quasi-simultaneous impedance spectra measurements of 24 sequentially switched sensors in the frequency range from 100 mHz to 100 kHz in approximately 30-min intervals.

Prior to the experiments, the measuring head was sterilized with alcohol-based chemicals approximately 24 h before performing the experiments. Immediately before installing the sensors, the system was sterilized in a laminar chamber using ultraviolet for about 10 min. The casings were removed from the QTF before the experiment and the sensors were rinsed with isopropanol (Avantor Performance Materials, Gliwice, Poland), which was evaporated at room temperature. A plate with bacterial cultures was prepared for the ready set of 24 sensors. Refreshed bacterial cultures were diluted in TSB to an OD_550_ (optical density) equal to 0.2. The culture of 10^6^ CFU/mL was suspended in 1.5 mL in 24 wells culture plate (VWR International, LLC Radnor Corporate Centre, Radnor, PA, USA) and incubated at 37 °C for 240 h. *Pseudomonas* phages LUZ19 filtrate at MOI 0.1, 1 or 10 was added at 6, 48 and 72 h of incubation. Each experiment was performed in triplicate with four technical repetitions (*N* = 12).

### 2.5. Impedance Spectra Analysis

The electrical impedance Z=R+jX expressed in ohms (Ω) is the complex measure of the object’s electrical response to the alternating voltage excitation, where R is a resistance, X is a reactance and j is the imaginary unit. The technique in which impedance spectra, that is the representation of such responses measured over a wide range of frequencies, is called the impedance spectroscopy [20]. Results of such a measurement may be also presented as the complex admittance Y expressed in siemens (S), where real and imaginary parts are conductance G and susceptance B. 

**Figure 2:**
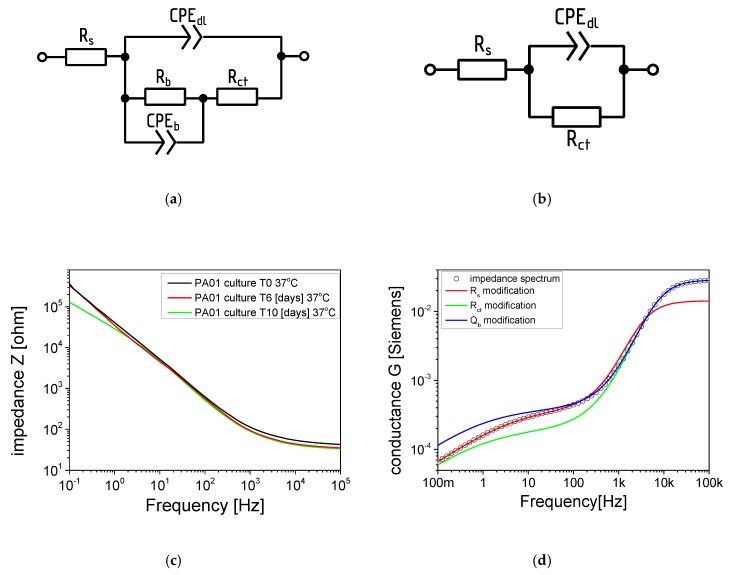
Primary (**a**) and simplified (**b**) electric equivalent circuits (EECs) used for the analysis of measured impedance spectra; (**c**) impedance spectra of quartz tuning forks (QTF) in *P. aeruginosa* PAO1 10-days biofilm culture, and the influence of EEC parameters change to the shape of conductance spectrum (**d**).

A typical impedance spectra analysis method is fitting them with the impedance of the electric equivalent circuit (EEC) using dedicated software (for example, Scribner ZView that was used in presented research). Two EECs were used (Figure 2a,b). 

These consisted of resistors and constant phase elements (CPE). CPE is the element frequently used in EEC modelling. Its impedance is equal $Z_{CPE}=\frac{1}{Q{\left(2\pi jf\right)}^{T}}$, where Q and T are CPE parameters and f is frequency. Its components relate to: Rs, series resistance which corresponds mainly to the growth medium resistance; CPEdl, electric double layer capacitance between the growth medium and electrodes; Rct, charge transfer resistance; Rb and CPEb, resistance and capacitance of the objects adhered to the electrode surface (mostly cells and biofilm). 

Most of the fits were done with primary EEC however, in some experiments, the influence of Rb and CPEb on the measurement spectrum was not measurable. In these cases the simplified EEC gave better-fitting results. The optimal model was chosen based on the fit quality (χ^2^) and the fitting error of EEC components. In simplified analyses after EEC modelling, the use of Rs and Qb parameters was proposed. Biologically, the changes in Rs parameter should be understood as an increase in the number of planktonic forms in the medium surrounding the QTF sensor [18]. The second important parameter of the EEC model is Q_b_ (electrical conductivity) measured at 100 mHz. The biological equivalent of changes for the conductance (Q_b_) is the adhesion of bacterial cells to the sensor surface as well as the formation or degradation of the biofilm matrix. The analysis of the frequency ranges at which the most important EEC components influence the impedance spectrum shape allowed a simplification of the biofilm state assessment. The plot shown in Figure 2d was created by simulating the conductance spectrum of the EEC fitted to one of the preliminary results (dots) and with R_s_, R_ct_ and Q_b_ changed twofold. As may be observed, a change in R_s_ altered the high-frequency part of the spectrum while Q_b_ influences the low-frequency part. Therefore the Rs variations may be estimated by the real part of the impedance measured at 100 kHz. As the initial value of such a parameter varied between sensors it was normalized by its value at the 3rd h of the experiment yielding R_100k_norm_ presented in the Results section. Similarly, the Q_b_ variations were estimated by the variations of the conductance (that is the real part of the inverse of the impedance) G_100m_ measured at 100 mHz. The similarity between parameters obtained using the EEC and simplified analysis is shown in Figure 6a,b. 

### 2.6. Biofilm Monitoring by Scanning Electron Microscopy (SEM) 

Sensors covered with bacterial biofilm were transferred to eppendorf tubes with PS to remove unadhered cells. Next, the sensors were placed in a series of increasing ethanol concentrations: 60%, 70%, 80%, 96% and the remaining water content was evaporated in vacuum conditions. After 24 h the preparations were transferred to a vacuum evaporator and the silver conductive layer spraying process was carried out. Biofilm presence on QTF surface was visualized by SEM at 30×, 100× and 3000× magnifications (Tesla BS 300, Brno, Czechoslovakia).

### 2.7. Statistical Analyses

The statistical analysis was performed for three independent experiments and four technical repetitions (*N* = 12) using a one-way ANOVA. To compare the differences between variances, Levene’s statistical method was applied. Results were considered significant at *p* < 0.05 value.

## 3. Results

### 3.1. Biofilm and Planktonic Population Growth Measured by Standard Colony Count and SEM 

**Figure 3:**
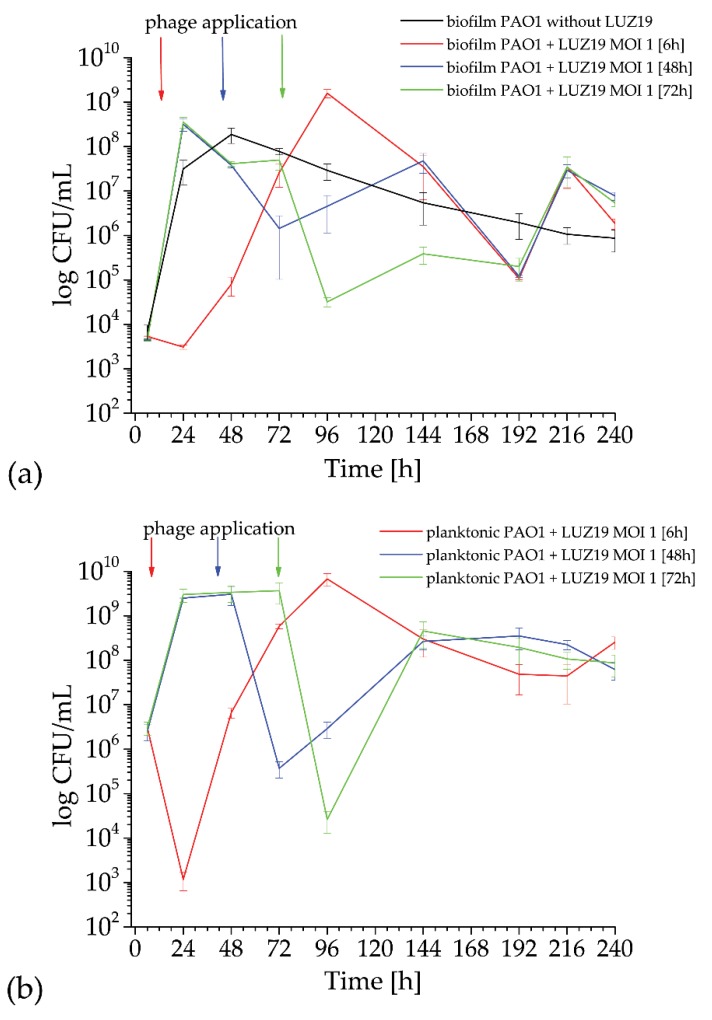
The colony count of *P. aeruginosa* PAO1 biofilm growth on QTF surface (**a**) and planktonic population (**b**) after phage LUZ19 infection. The error bars indicate the standard deviation. The results are presented as the means CFU/mL ± SD of three independent experiments in triplicate.

The dynamics of PAO1 biofilm growth on quartz surface was determined by a standard colony count for both phage treated and untreated culture (Figure 3). The phage LUZ19 at MOI = 1 was applied at three different culture times (marked with colored arrows in Figure 3), corresponding to the 6th, 48th and 72nd h of incubation. Cell counts were measured starting from 6 h until 10 days of incubation. In the untreated biofilm experiment, an increase in the number of cells is observed, with a maximum of about 10^8^ CFU/mL for 48 h incubation (black curve in Figure 3a). Further incubation gradually reduces the population up to around 10^6^ CFU/mL on the tenth day of the experiment. Phage application at the 6th h inhibited the propagation of sessile cells for one day, and further, the biofilm population started growing up to the maximum of 10^9^ CFU/mL after four days of incubation (red curve in Figure 3a). The application of phage LUZ19 at MOI = 1 at 48 and 72 h (blue and green curves in Figure 3a) caused a decrease in the number of sessile cells at the level of 2–3 logs followed by the population regrowth and a final count of around 10^6^ CFU/mL. The permanent exposition of biofilm to phage infection resulted in the fluctuation of the cell number embedded in biofilm matrix from 10^9^–10^8^ CFU/mL to 10^5^ CFU/mL. Prolonged biofilm cultures infected with phage LUZ19 stabilized the colony count at around 10^6^ CFU/mL after 10 days of incubation regardless of the time point of phage infection. 

LUZ19 phage infection at the beginning of planktonic culture incubation (6 h) caused the drop of 3 logs when measured at a 24th of the incubation time. The next day, a clear regrowth of planktonic forms to the level of 10^7^ CFU/mL was observed and gradually increased up to a maximum of 10^9^ CFU/mL at the 96th h of the experiment. A similar dynamic was noticed for planktonic forms, where the phage was applied at the second and third day of incubation, leading to a sudden drop of 3–4 logs in CFU after one day of phage propagation forwarded by the fast phage-insensitive population regrowth with the maximum of 10^8^ CFU/mL (blue and green curve in Figure 3b). All phage-treated planktonic cultures stabilized the colony count at around 10^8^ CFU/mL after 10 days of incubation. 

**Figure 4:**
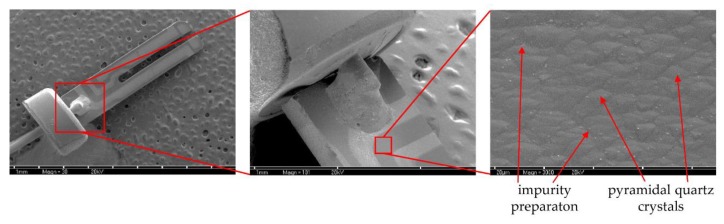
Visualization of QTF sensor surface immersed in TSB medium (without bacterial cells) at magnifications of 30× (left part) 100× (middle) and 3000× (right part), respectively.

To confirm the presence of biofilm formation on the surface of the QTF sensor, we performed the analysis using a scanning electron microscope (SEM). As part of the control experiment, the sensors were suspended in TSB broth without bacteria (Figure 4). Microscopic images show the structure of the sensor surface. It consists of evenly distributed quartz crystals making a pyramidal form. Bright elements appearing are not bacterial cells but likely derive from diluents or result from post-production sensor impurities. 

**Figure 5:**
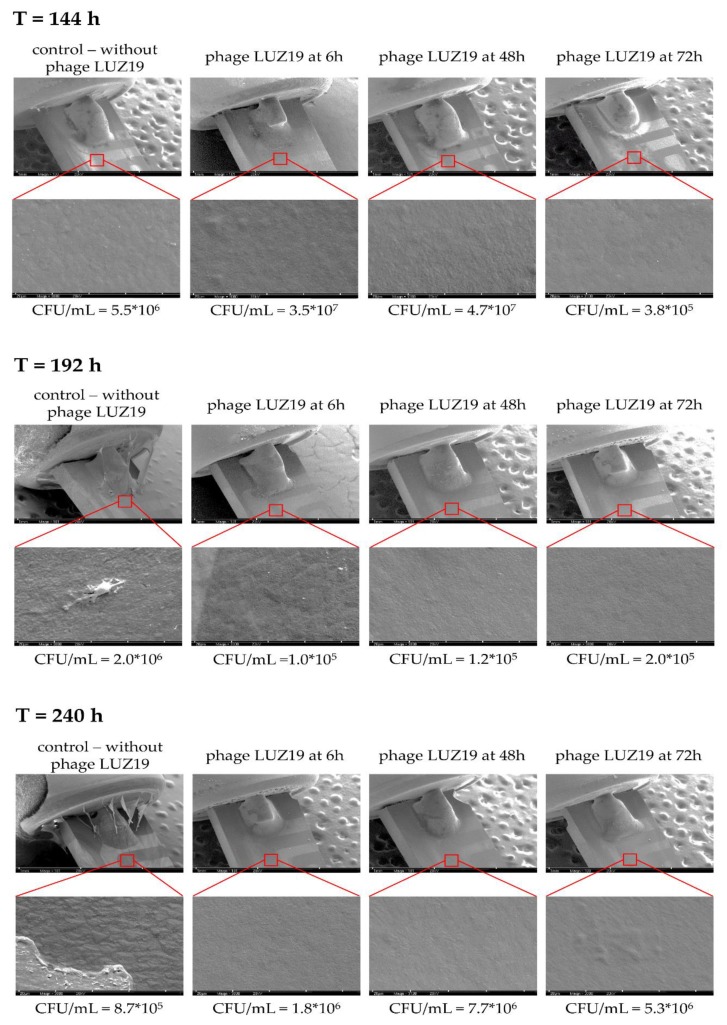
Visualization of QTF sensors at the 144th, 192nd, and 240th h of untreated and phage LUZ19 treated biofilm at MOI = 1 (from left to right, added at the 6th, 48th and 72nd h of culture, respectively). The upper panel shows the tuning fork ring at 30× magnification. The bottom panel enlarged 3000× QTF surface. The bottom panel presents the CFU/mL values corresponding to selected time point.

Time points of biofilm visualization with SEM (Figure 5) were selected based on changes in the G_100m_ parameter (see paragraph 3.2). Figure 5 is divided into upper and lower panels, and sensors are presented with untreated biofilm after phage LUZ19 application at the 6th, 48th and 72th h of incubation. The upper panel shows the tuning fork ring at 30× magnification, while the lower panel shows the QTF quartz surface at 3000× magnification. Under the panel, the CFU/mL values corresponding to selected time points are added. In the case of QTF incubated in phage untreated PAO1 culture, the pyramidal quartz crystals are completely obscured by biofilm components and the entire surface is covered by a layer of EPS matrix, and EPS matrix fragments are clearly visible on days 8 (196 h) and 10 (240 h) of biofilm incubation. The matrix and densely arranged bacterial cells in anchored large groups are visible at a higher magnification. At the end of the experiment (240 h), the microscopic visualization shows a reduction in EPS thickness and cells are no longer fully covered with a thick matrix layer. In contrast, there were no significant changes in SEM pictures between phage-treated biofilm and only a thin EPS layer was detected for all samples. The differences in the amount of EPS/matrix covering the sensor surface were seen for 196 h and 240 h biofilm compared to untreated samples. Considering the results of the colony count and SEM analyses, it might be assumed that the differences in biofilm imaging seen in microscopy analysis are rather related to the amount of the EPS and not to the number of sessile cells embedded in biofilm structure. 

### 3.2. Standarization of Real-Time Monitoring of P. aeruginosa PAO1 Biofilm Growth under Control Conditions Using QTF Impedance Spectroscopy

**Figure 6:**
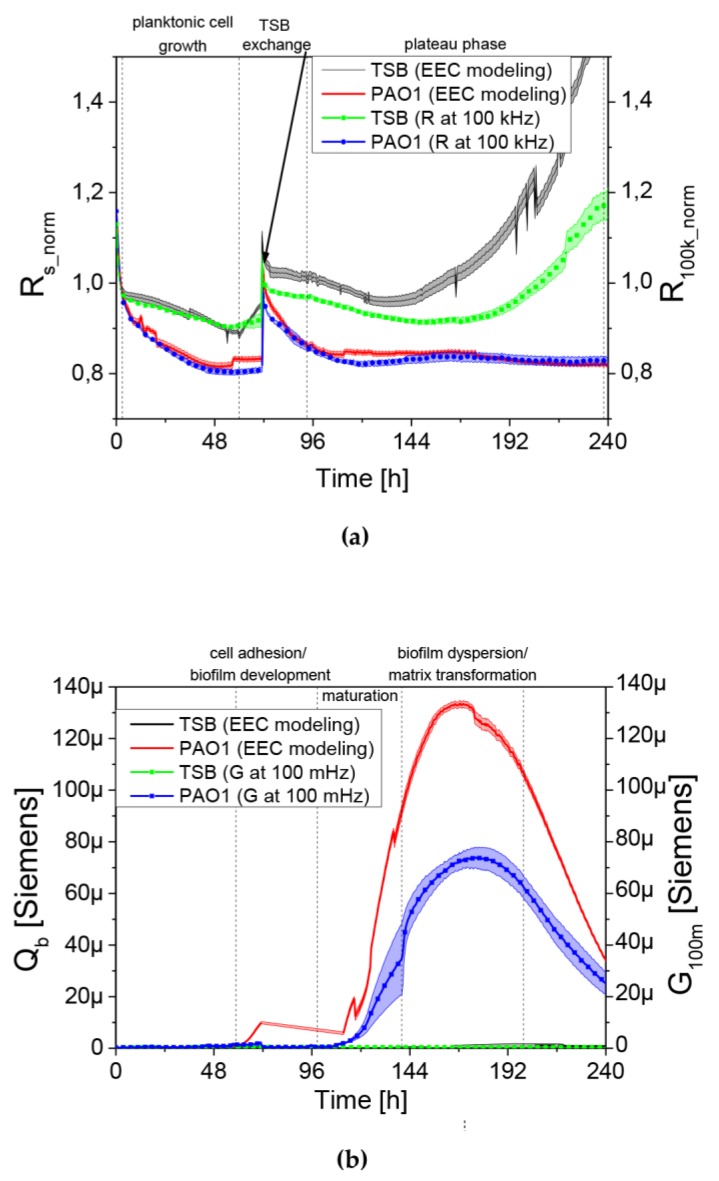
(**a**) Comparison of the normalized resistance parameter measured by the method using EEC model (parameter R_s_norm_—left axis) with the measurement at one frequency of 100 kHz (parameter R_100k_norm_—right axis); (**b**) Comparison of the conductance parameter for the method using EEC model (parameter *Qb*—left axis) the measurement at 100 mHz (parameter G_100m_—right axis).

The standardization of a real-time monitoring of *P. aeruginosa* PAO1 biofilm growth using QTF impedance spectroscopy was performed (Figure 6). The initial drop of resistance R_100k_ was caused by the temperature stabilization of growth medium. 

Starting from the sixth h of the experiment, the R_100k_ value was gradually decreasing for up to 48 h for the sensor immersed in bacterial culture compared to the TSB control. This change is connected to bacteria growth near the sensor surface. After transferring to a new TSB medium at 72 h of incubation, there was a peak in the resistance value as the result of the stabilization of the sensor in a new medium followed again by the planktonic forms propagation until a stable level of R_100k_norm_ was seen at the same level as before the exchange of medium (~110–120 h). On the fourth day of the experiment, R_100k_norm_ parameter reached a plateau that it maintained until the end of experiment. For QTF sensors immersed in TSB medium, a temperature stabilization of about three h was observed. Next, the sensor was functionalized and metallic electrodes underwent an oxidation process which is expressed by a slight decrease in the R_100k_norm_ parameter. The sensor transfer into fresh medium at 72 h caused a similar peak formation as that for the tested sample. At the later stages in the TSB control experiment (after 144 h), an increase in resistance was observed over time, which can be equated with the physicochemical changes of the medium and the further oxidation processes on the QTF surface. The second measured parameter (Q_b_) was used to assess the electrical conductivity of biological objects adhering to the sensor surface. Fresh medium introduction at 72 h lead again to Q_b_ plateau until ~110–120 h. There was a relationship between the resistance parameter (R_s_norm_) and Q_b_ representing the conductance parameter. After reaching the plateau level in Rs, the electrical conductivity Q_b_ was increasing. The Q_b_ grew from ~110–120 h to ~ 180 h of experiment, reaching the value of 130 μSiemens. That increase can be explained by bacterial cell adhesion to the sensor surface and by formation of the conductive EPS matrix. After 180 h of incubation, the Q_b_ curve was going down and those changes were identified as the dispersion of biofilm or modifications and degradation of matrix presented as the depletion of electrical charge in the broth culture. Later in the text, the Q_b_ parameter is replaced by a G_100m_ conductance parameter derived for simplified EEC as explained in the Materials and Methods Section 2.5. The dynamics of conductance changes in G_100m_ identified with the creation and reconstruction of EPS matrix, requiring the need for visualization using a scanning microscope, which directly proved the correlation of the electrical parameter with the IS method and visualization in SEM. Conductance changes in G_100m_ have been shown to be strongly associated with the reduction of EPS matrix in longer biofilm cultures.

### 3.3. Monitoring of PAO1 Biofilm Growth during Pseudomonas Phage LUZ19 Treatment Measured by Impedance Spectroscopy

**Figure 7:**
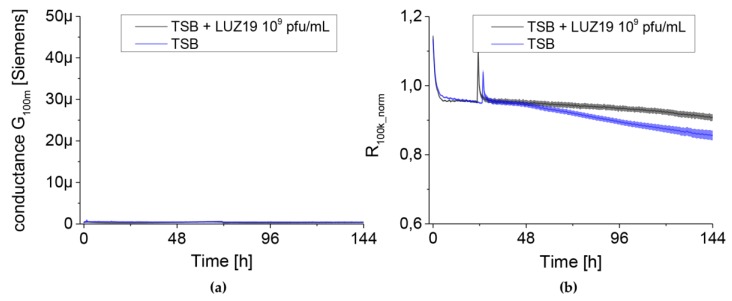
The changes in conductance G_100m_ (left panel) and resistance R_100k_norm_ (right panel) measured by QTF sensors for negative control consisted of phage LUZ19 and TSB medium. Data are expressed as mean conductance G_100m_ ± SD and R_100k_norm_ of four independent experiments.

All control experiments, by standardizing experimental conditions, enabled the application of impedance measurements using the QTF sensor platform to monitor the dynamics of biofilm growth during the treatment with phage LUZ19. Additional negative controls consisted of 10^9^ pfu/mL of phage LUZ19 in TSB medium (Figure 7).

The measured parameters of normalized resistance at 100 kHz for TSB curves and phage LUZ19 ran similarly over the course of the seven-day experiment. For the G_100m_ parameter, no conductivity disturbances of QTF sensors were also observed, which confirmed the possibility of biofilm dynamics measurement [18]. For biofilm treatment, phage LUZ19 was used at three MOIs (0.1, 1 and 10) and different times of administration. The MOI value was calculated based on the CFU value presented in Figure 3. The phage application points corresponded to the 6th, 48th and 72nd h of culture incubation to obtain the effect of adhesion prevention and biofilm eradication at different time points. The experiments were carried out for 10-days in the culture. Two electrical parameters of the culture were measured in real-time to determine biofilm formation dynamics. The first parameter was the conductance G_100m_ associated with bacterial cell attachment to the surface of the QTF sensor and the growth of sessile forms. In parallel, the series resistance (Rs) of the medium showed the changes in the medium composition and planktonic cells growth. The dynamics of electrical parameters of PAO1 biofilm culture treated with phage LUZ19 at different MOIs were compared to a non-infected control. 

**Figure 8:**
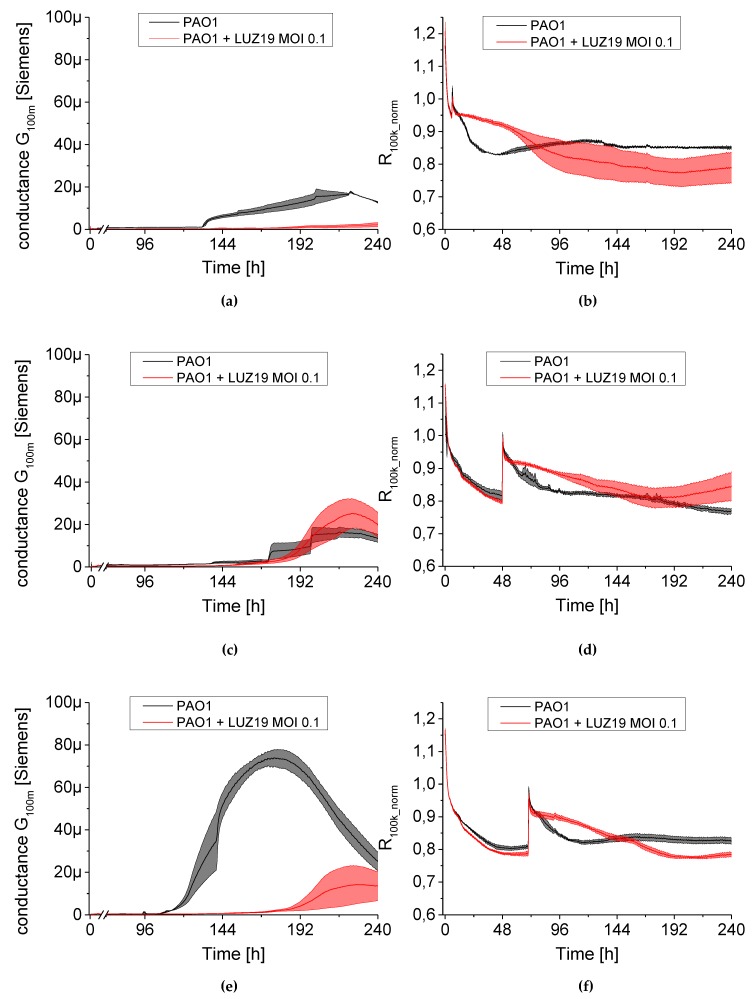
The changes in conductance G_100m_ (left panel) and resistance R_100k_norm_ (right panel) measured by QTF sensors during phage LUZ19 treatment of PAO1 biofilm at MOI = 0.1. Phage was applied at the 6th (**a**,**b**), 48th (**c**,**d**) and 72nd (**e**,**f**) hour of biofilm culture. In the right panel, for each variant, a peak appears, which is associated with the transfer of QTF sensors to new medium containing an appropriate concentration of viral particles. Data are expressed as mean of conductance G_100m_ ± SD and resistance R_100k_norm_ from three independent experiments.

**Figure 9:**
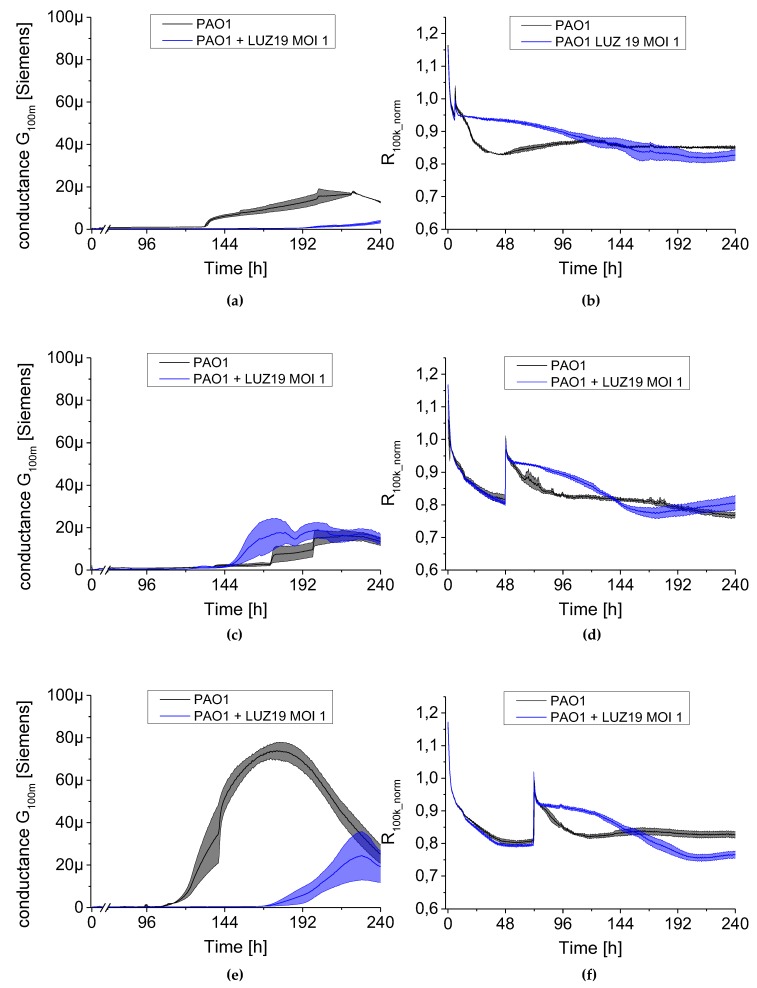
The changes in conductance G100m (left panel) and resistance R100k_norm (right panel) measured by QTF sensors during phage LUZ19 treatment of PAO1 biofilm at MOI = 1. Phage was applied at the 6th (**a**,**b**), 48th (**c**,**d**) and 72nd (**e**,**f**) hour of biofilm culture. In the right panel, for each variant, a peak appears, which is associated with the transfer of QTF sensors to new medium containing an appropriate concentration of viral particles. Data are expressed as mean of conductance G100m ± SD and resistance R100k_norm from three independent experiments.

**Figure 10:**
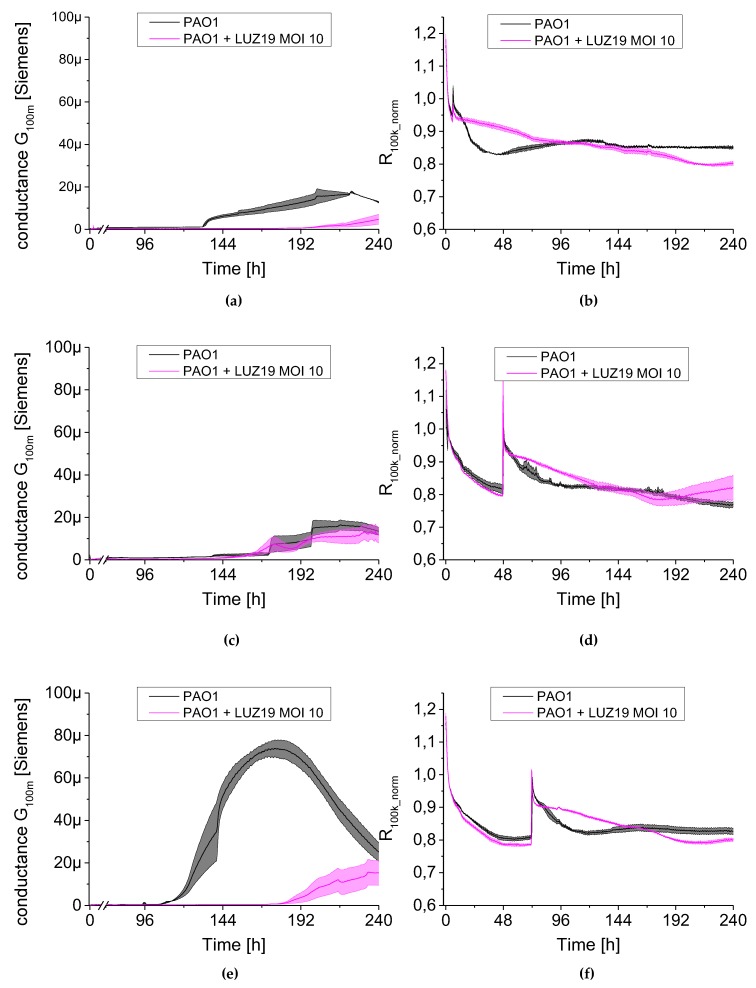
The changes in conductance G_100m_ (left panel) and resistance R_100k_norm_ (right panel) measured by QTF sensors during phage LUZ19 treatment of PAO1 biofilm at MOI = 10. Phage was applied at the 6th (**a**,**b**), 48th (**c**,**d**) and 72nd (**e**,**f**) hour of biofilm culture. In the right panel, for each variant, a peak appears, which is associated with the transfer of QTF sensors to new medium containing an appropriate concentration of viral particles. Data are expressed as mean of conductance G_100m_ ± SD and resistance R_100k_norm_ from three independent experiments.

The results are presented as conductance (left panel Figure 8, Figure 9 and Figure 10) and normalized series resistance (right panel Figure 8, Figure 9 and Figure 10). A set of 24 sensors covered by bacterial biofilm was transferred at selected time points to a new plate containing TSB broth with phage LUZ19 suspended at appropriate MOIs. The control non-treated PAO1 culture was also transferred to new TSB medium simultaneously as the treated sample. The dynamics of the G_100m_ conductance parameter changed after phage application (Figure 8, Figure 9 and Figure 10, left panels) and were very similar regardless of MOI applied. We noticed that the moment of phage administration largely determined the course of curves for measuring electrical parameters. 

When phage was administered at six h of culture incubation, a strong inhibition in biofilm growth was observed regardless of MOI applied (Figure 8a, Figure 9a and Figure 10a). The G_100m_ parameter for PAO1 control began to increase at around 100 h into the experiment, reaching 20 μSiemens after 220 h, while the conductance of phage-infected culture was at 3–5 μSiemens up to 10 days into the experiment. The propagation of planktonic cells was slowed down when phage was applied at six h of culture incubation (Figure 8b, Figure 9b and Figure 10b). The resistance parameter (R_100k_norm_) made up the control culture at around the 60th and the 100th h of incubation for MOI 0.1 and MOI 1–10, respectively. Other results were obtained after LUZ19 application at 48 h of the culture. Biofilm was not sensitive to phage therapy regardless of applied phage concentration in the culture medium. The maximum conductance was observed around 220, 168 and 192 for MOI 0.1, 1 and 10, respectively, corresponding to the conductivity of around 20 μSiemens. The analysis of planktonic forms via serial resistance R_100k_norm_ showed similar dynamics in generating phage-resistant forms regardless of MOI. The biofilm infection at 48 h after the start of the experiment generated similar cell regrowth regardless of applied LUZ19 concentrations, reaching a plateau phase by about the 144th h. 

There was a delay in biofilm formation compared to the control PAO1 when phage was introduced at the 72nd h. The conductance dynamics was MOI independent, reaching the maximum of 30 μSiemens. The biofilm cultures on the sensors were carried out in stationary conditions before being transferred to the medium containing phage LUZ19. It is likely that the biofilm EPS at 72-h biofilm differs in the composition compared to the 48-h one, making the sessile cells sensitive to phage exposition. In Figure 8e, Figure 9e and Figure 10e a shift in the conductivity G_100m_ increase was observed and the maximum was reached at about 230 h, for each MOI. In this case, very similar fluctuations were found within the parameter characterizing planktonic forms. The R_100k_norm_ curves reached a plateau phase of about 192 h for each MOI tested (Figure 8f, Figure 9f and Figure 10f).

## 4. Discussion

Our previous experience with the application QTF sensor platform regarded biofilm biomass measurements when *P. aeruginosa* sessile cells were treated with antibiotics [15,21]. The changes in the resonant frequency of QTFs covered with biofilm structure were measured, which were then converted into the mass of bacterial biofilm at selected culture time points. That technology was limited to particular incubation times and it required complex material preparation procedures. In the presented study, we were looking for real-time measurement techniques remotely monitoring the biofilm formation and degradation. We have shown that the construction and arrangement of electrical covers of QTF allow for its application as an impedance spectroscopy sensor [18]. The applied impedance spectroscopy (IS) technique using QTF sensors is a convenient technology for biofilm growth monitoring in a real-time manner. IS was commonly used to determine the behavior of materials in chemical systems. It has become a tool for chemical and electrochemical analysis of materials with different levels of conductivity [21]. It has also found an application in the broadly understood biological sciences. Measurements of electrical parameters of biological objects have been monitored since around 1970, mainly to determine food contamination by bacteria [22]. Impedance spectroscopy is becoming widely used in experiments with bacterial biofilms as described by the Paredes group or van Duuren team [23,24]. Usually, these experiments base their measurements on a commercial instrument, the xCELLigence Real-Time Cell Analyzer (RTCA) from Acea Biosciences that measures impedance in 96 well plates equipped with gold microelectrodes by measuring the so-called cell index for *Pseudomonas* and *Streptococcal* biofilms [24,25]. Our previous experience also showed that IS using QTF is an effective technique for non-invasive analysis of biofilm culture parameters [18] extending now the aspect of the phage application model. The sensors used are planar and often the electrodes are finger-shaped. QTF sensor design is very convenient for spectra characterization and the creation of an electric equivalent model. In our technology, QTF sensors are immersed in bacterial culture enabling bacterial cells adhesion and an increase of biofilm monitoring. We also use a neutral metallization for tested objects, because QTF is mainly made of quartz and aluminum. The materials that make up the sensor are also relatively inexpensive, which is why the technology we propose is a relatively cheap tool for the monitoring of bacterial biofilm physiology. 

The results obtained in our study are presented as simplified analyses measured in the form of conductance and resistance. Due to differences in the quality of QTF sensors, their surface functionalization in the culture medium and stabilization of temperature conditions (heating the TSB medium to 37 °C), the results for series resistance (R_100k_) were normalized to three h of experiment and presented as R_100k_norm_. Compared to commonly used methods, the undoubted advantage of IS based on QTF is the ability to measure parameters characterizing simultaneously the changes in planktonic forms in the culture medium and the biofilm formed on the sensor surface (parameters R_100k_norm_ and G_100m,_ respectively). The dynamics of both parameters resulted from the equivalent electrical circuit (EEC) model we had previously set up and verified. The impedance spectroscopy measurements are highly repeatable at a high resolution. The spectra collection took place for the same sensor every 30 min. This allowed us to obtain reliable results, without the need for complex sample manipulation, as is the case with many recommended biofilm monitoring methods that have so far been described in *Crit Rev Microbiol* [13]. 

Most of the laboratory methods used to determine biofilm growth rely on the measurements at a given culture point. This applies to methods based on CFU/mL calculation, as well as staining techniques [26]. Moreover, these experiments are usually limited to several hours of incubation, and in many cases, we are not able to characterize the phenomenon of biofilm dynamic changes. In the case of IS using QTF, the experiments were carried out after up to 10 days of incubation. Our measurements were carried out semi-parallel to each sensor. The built platform allowed 24 independent experiments to be performed simultaneously, while the entire measurement took place in a titration plate in batch culture. 

We observed a clear relationship between the series resistance parameter (R_100k_norm_) and conductance (G_100m_). When R_100k_norm_ resistance reached the plateau phase, G_100m_ conductance increased significantly. The lowering resistance is associated with an increase in planktonic cell number in the medium (described earlier in [18]). The increase in the conductance is identified with the appearance and level of surface coverage by a conductive EPS matrix. Based on the G_100m_ parameter, we measured the QTF coverage with an EPS matrix that changes the charge transfer across the barrier between the electrode and electrolyte. However, the drop in the conductance parameter for G_100m_ is associated with a reduction of EPS or a change in the physicochemical properties of the biofilm matrix. The culture was carried out in stationary conditions, without medium exchange, therefore, the conductance reduction scenario was associated with the reuse of matrix components as an energy source of bacterial culture. The SEM visualization of QTF surface on the 6th, 8th and 10th day of biofilm formation combined with the colony count results provide the proof that the above conclusions were correct.

We used a QTF as an impedance sensor to measure electrical changes of PAO1 culture during phage LUZ19 infection at three different MOIs corresponding to 0.1, 1 and 10. An inhibitory effect on biofilm formation was seen when the infection took place at the 6th and 72nd h of incubation, regardless of MOI applied. It has also been shown that generally, the biofilm dynamics were independent of phage LUZ19 infecting dose. Interestingly, 48-h biofilm turned out to be a breakthrough moment, because at this time phage administration did not affect the biofilm formation, although the killing influence on both sessile and planktonic cells were recorded. Comparing the results from three different biofilm monitoring techniques (colony count, SEM, and impedance), we may conclude that G100m parameter shows, in fact, the amount of the biofilm EPS accumulating in the sensor surface. 

The impedance spectroscopy model we designed is able to monitor the behavior of planktonic culture surrounding the QTF sensor. The R_100k_norm_ parameter indicating the culture propagation dynamics in the case of phage infection means the inhibition of phage-sensitive cell growth and secondary regrowth events of selected phage resistant bacteria. That population regrowth and dynamic phage–bacteria balance in the biofilm structure was confirmed in the colony count detection.

The application of *Pseudomonas* phages in order to eradicate bacterial biofilm is widely described in the literature showing both successes and failures. Some phages like phage E27 or M4, are not able to penetrate the biofilm matrix, thus, they are ineffective in biofilm elimination [27,28]. The strong restriction of phage access to cells embedded in the dense biofilm EPS matrix can be partially overcome by virion-associated depolymerases produced by some phages such as LUZ19, tested in the presented study [29]. Phage infection usually led to a significant cell number reduction (3 logs) on catheter surfaces but the incomplete eradication resulted in a fast population regrowth within the next 24 h [28]. Because of the presence of phage insensitive portions in the sessile cells, the idea of combined phage-antibiotic treatment was also studied [30]. The synergy effect of podovirus phage LUZ7 and streptomycin was noticed against *P. aeruginosa* PAO1 biofilm compared to a single preparation [30]. A similar synergy observation was done by the Knezevic group [31]. Nevertheless, the heterogenic character of the sessile population protects the biofilm from a complete elimination regardless of an applied anti-biofilm agent. The limitation in the biofilm eradication is also caused by rapid emergence of phage-resistant phenotypes [32,33]. 

As phages are co-evolving with its host, there are some contrary mechanisms of phage influence on biofilm formation and maintenance. This specifically concerns prophages having a huge impact on bacterial phenotypes. It is already proven that the remodeling of a biofilm EPS matrix and the development of small-colony phenotypic variants are associated with the presence of filamentous prophage Pf4 (Pf) in *P. aeruginosa* genome [34]. It turns out that Pf participates in the organization of biofilm matrix, while being induced, release and transform into crystal lattices. This modification of matrix improves the adhesion process and increases drying and antibiotics tolerance [34]. Tobramycin applied with the biofilm treatment accumulates inside liquid crystals of Pf phage particles, whereas a strong packaging of negatively polarized phage polymers interact with positively charged antibiotics [35]. Pf phage activity is not only limited to the modification of a specific host EPS matrix, but may affect the biofilm growth of distant accompanying organisms (*Aspergillus fumigatus)*, by direct capture of Fe^3+^ ions (and other multivalent metals) [36,37]. Above prophage activity is a newly discovered mechanism of restrictive regulation of the biofilms especially in *P. aeruginosa* strains associated with cystic fibrosis patient infection. 

In conclusion, the technology we presented in this study gives a reproducible and precise measurement of biofilm structure and planktonic culture dynamics analyzed simultaneously and in a real-time manner without specific sample preparation. The impedance parameters such as the conductance and resistance of biological samples give more insight into the bacterial culture changes happening during the natural growth and the fluctuation of culture characteristics when treated with antibacterial agents. Moreover, the impedance parameters turned out to be specific for EPS amount measurement. Therefore this system seems to be very useful in the monitoring of antibacterial effects in biofilm prevention and eradication, and especially for the study on phage influence on biofilm matrix development.

## Abbreviations

**QTF**	Quartz Tuning Fork
**G_100m_**	conductance of simple analysis measured at frequency 100 mHz
**R_100k_norm_**	normalized resistance of simple analysis measured at frequency 100 kHz
**EPS**	extracellular polymeric substances
**IS**	impedance spectroscopy
**Z**	impedance module
**G**	conductance
**TSB**	tryptic soy broth
